# Prognostic value of cGAS-STING-IRF3 signaling in cholangiocarcinoma patients

**DOI:** 10.1371/journal.pone.0342756

**Published:** 2026-02-18

**Authors:** Parawee Artbua, Naruemon Kentachalee, Sirinya Sitthirak, Prakasit Sa-Ngiamwibool, Phongsathorn Wichian, Raksawan Deenonpoe

**Affiliations:** 1 Department of Pathology, Faculty of Medicine, Khon Kaen University, Khon Kaen, Thailand; 2 School of Allied Health Sciences, Walailak University, Nakorn Si Thamarat, Thailand; 3 Cholangiocarcinoma Research Institute, Khon Kaen University, Khon Kaen, Thailand; Northwest University, UNITED STATES OF AMERICA

## Abstract

Cholangiocarcinoma (CCA) is an aggressive malignancy with a poor prognosis, often diagnosed at an advanced stage. Chromosomal instability (CIN), a hallmark of cancer, leads to the release of cytosolic double-stranded DNA (dsDNA), which activates the cGAS-STING pathway and its downstream immune signaling. However, the prognostic implications of this pathway in CCA remain poorly understood. This study aims to examine the cGAS-STING pathway-related proteins in CCA and their correlation with clinicopathological parameters. A total of 164 formalin-fixed paraffin-embedded (FFPE) CCA tissue samples were analyzed using tissue microarray (TMA) and immunohistochemistry (IHC). Statistical analysis assessed correlations between proteins expression and clinicopathological features were assessed using Chi-square tests, logistic regression, Kaplan–Meier survival analysis, Cox proportional hazards models, and Spearman’s rank correlation coefficient. Moderate–to–high STING expression was significantly associated with reduced tumor size and lymphovascular invasion but paradoxically correlated with short overall survival (p < 0.05). In contrast, moderate–to–high γH2AX expression predicted improved survival. IRF3 expression was significantly higher in the tubular histological subtype of CCA compared to the papillary subtype (p = 0.012), indicating a possible morphological correlation. Multivariate analysis confirmed STING as an independent prognostic marker for CCA. Our findings suggest that STING appears to function as a double-edged sword in CCA, limiting local invasion while paradoxically contributing to poor survival outcomes. IRF3 expression appears linked to histological subtypes, supporting its role in tumor biology. These markers may provide valuable insights into tumor behavior and may guide treatment strategies in CCA patients.

## Introduction

Cholangiocarcinoma (CCA) is an invasive type of cancer originating from the bile duct epithelial cells. The prognosis of cholangiocarcinoma patients is poor; it is a type of liver cancer that does not develop in the same way as other types of liver cancer and is often confused with other liver cancers. However, bile duct cancer arises from the lining of the biliary tract, a disease that typically has no symptoms in its early stages. CCA is mostly detected once the cancer has already developed [1]. One of the key hallmarks is chromosomal instability (CIN), which is an increased rate of chromosomal segregation abnormalities and cancer progression involving genetic alterations [2]. This contributes to DNA damage caused by multiple genes that regulate cell division and tumor suppression [3]. CIN is scrutinized as a prognostic factor in cancer, and its presence may influence the response to certain cancer treatments. Molecular testing of a CCA patient, including assessments for CIN, is often part of the cancer diagnosis and treatment planning. CIN promotes inflammatory signaling by introducing double-stranded DNA (dsDNA) into the cytoplasm, which results in the activation of γH2AX, a key marker of DNA repair and damage [4], as well as a sensitive marker of DNA double-strand breaks (DSBs). DSBs may lead to cancer, but conversely, DSBs are also used to kill cancer cells, engaging the primary source [5]. The cGAS-STING pathway is activated by cytosolic dsDNA, often introduced by chromosomal instability, leading to micronuclei formation [6]. Micronuclei are small, membrane-bounded compartments with DNA content encapsulated by a nucleus separated from the primary nucleus [7]. The protein cGAS or cyclic GMP-AMP synthase, is an important component of the innate immune system and acts as a secondary messenger for processes such as cell proliferation, cell death, immune response, and inflammation. This protein plays a crucial role in detecting the presence of cytosolic dsDNA [8]. c-GAS is one of those activated by DNA damage. This stimulus, known as damage-associated molecular patterns (DAMPs), is one of the innate immune systems and can stimulate a line of defense against invading pathogens. It detects pathogen-associated molecular patterns (PAMPs) as well [9]. The Stimulator of Interferon Genes (STING) is a critical adaptor protein in the cGAS-STING pathway. Once activated, it leads to downstream signaling, activating transcription factors such as Interferon Regulatory Factor 3 (IRF3) and Nuclear Factor kappa-light-chain-enhancer of activated B cells (NF-kB). This activation produces type I interferons and other inflammatory cytokines, contributing to antiviral defense and immune regulation [10]. After cGAS binds STING IRF3 is triggered to move into the nucleus, stimulating the production of interferon-alpha (IFN-α), which helps coordinate the immune response against cancer. This pathway plays an important role in cancer treatment. The cGAS-STING pathway affects most human cancers between the cancer process and inflammation. When the body responds to injury normally, many systems control the wound-healing processes [11]. Moreover, chromosome abnormalities also impact the course of the disease progression in CCA patients, such as trisomy of chromosomes 7 and 17, which are associated with poor prognosis and metastasis in CCA patients, indicating CIN [12]. A new cancer therapy, amido benzimidazole (ABZI), acts as an agonist for the cGAS-STING system, which is involved in the body’s immune response by controlling the structure of proteins that resist pathogens and regulate cancer cell growth [13]. A recent study on pancreatic cancer, which shares precursors with CCA, demonstrated the use of the cGAS-STING pathway to promote T cell activation that is toxic to cancer cells. This has potential for combination therapies in immunotherapy [14]. In previous reports, higher cGAS expression is a prognostic factor with prolonged disease-free survival and overall survival [15]. This knowledge may contribute to developing therapeutic strategies for conditions related to immune system dysfunction. In recent years the effectiveness of these therapies has been particularly noted in tumors with high immunogenicity, such as melanoma. Cancer biotherapeutic research has been useful in CCA and liver cancer. To date, current immunotherapy for cancer includes vaccines, immunotherapy, gene therapy, and anti-angiogenic therapy [16].

To our knowledge, this study is among the first to investigate cGAS expression in CCA. This study aims to investigate the expression level of γH2AX, cGAS-STING, IRF3, and IFN-α in CCA patients and analyze their correlation with clinicopathological parameters. Using tissue microarray (TMA) in conjunction with the immunohistochemistry (IHC) technique were analyzed protein expression levels in formalin-fixed paraffin-embedded (FFPE) tissues. The correlation between cGAS-STING pathway-related protein expression and clinicopathological data in CCA patients may reveal prognostic factors that could inform treatment decisions and improve overall survival in CCA patients.

## Materials and methods

### Human ethics

The study was conducted on the Declaration of Helsinki and the International Council for Harmonization (ICH) Good Clinical Practice Guidelines, and the protocol (Approval No. HE671259) was approved by the Ethics Committee for Human Research, Khon Kaen University, Thailand. Formalin-fixed paraffin-embedded (FFPE) was used for immunohistochemical study. the consent form was not obtained due to the data being analyzed anonymously and its retrospective nature of the study and the use of anonymized archived specimens. The waiver of informed consent was approved by Khon Kaen University Ethics Committee for Human Research.

### Data collection

CCA FFPE tissue samples were accessed for research purposes on 01/07/2024. Tumor tissue samples from CCA patients were collected at Srinagarind Hospital, Khon Kaen University, Thailand. Clinical and demographic data—including gender, age, date of hepatectomy, and FFPE CCA tissues—were retrieved by the Cholangiocarcinoma Research Institute (CARI), Faculty of Medicine, Khon Kaen University, Thailand. These data were obtained from patients diagnosed with CCA between 01/01/2013 and 31/12/2022. Clinical data retrieval, FFPE block selection, and preparation of tissue microarrays (TMAs) for this study were conducted between 01/07/2024 and 17/01/2025. The study included 164 FFPE samples, comprising 116 males and 48 females. Clinicopathological data included tumor size, anatomical location, morphology, histological subtype, lymph node metastasis, lymphovascular invasion, perineural invasion, and overall survival.

### Tissue microarray (TMA)

Formalin-fixed paraffin-embedded tissues with 4-micron thickness and stained with hematoxylin and eosin (H&E) were selected in specific areas by pathologists. A tissue puncher with a 3 mm diameter was used to extract cylindrical punch tissue samples. The TMA sections were placed on glass slides, with three cores from one patient. The FFPE samples were re-embedded into recipient paraffin blocks for microarrays. Each slide contained 40 cores, including 36 CCA samples, 4 positive controls, and 1 negative control. A total of 14 TMA slides were used for each antibody, resulting in 70 TMA slides overall.

### Immunohistochemistry (IHC)

Immunohistochemistry (IHC) was performed at the Immunohistochemistry Unit of the Pathology Laboratory, Faculty of Medicine, Khon Kaen University. Tissue microarray (TMA) from a previously described cohort was prepared. After sectioning the TMA slides, it was baked overnight at 50°C. The baked TMA slides were then stained using the Ventana Benchmark XT automated stainer (Ventana Medical system, Arizona, USA). Slides were processed with 1X EZ Prep solution (supplied by Ventana Medical Systems, UK), for deparaffinization and rehydration. Cell Conditioning 1 (CC1- provided by Ventana Medical Systems, UK) was used for antigen retrieval to enhance antibody binding. The basic pH and tris-based buffer in CC1 enhanced antibody binding antigen (Ventana Medical Systems, UK) and used a cover to prevent the slide from drying out. Endogenous proteins and peroxides were blocked using Ventana diluent. Primary antibodies used included a rabbit polyclonal Gamma H2A.X antibody (phosphor S139) (γH2AFX; dilution 1:400, ab11174; Abcam), rabbit polyclonal anti-human c-GAS antibody (dilution 1:200, Proteintech, US), rabbit polyclonal anti-human STING antibody (dilution 1:3000, Proteintech, US), rabbit polyclonal anti-human IRF3 antibody (dilution 1:800, Proteintech, US), and rabbit polyclonal anti-human IFN alpha antibody (dilution 1:50, Proteintech, US). The immunohistochemical reaction was visualized using the Ultraview Universal Diaminobenzidine IHC Detection Kit (Ventana Medical Systems, UK). The slides were dehydrated using distilled water, followed by 95% and 100% ethanol, and xylene. Finally, the stained tissue microarray slides were scanned entirely to assign for image analysis and scoring.

### Image analysis

The staining results of IHC interpretation were performed by pathologists and compared using the open-source digital image analysis software, QuPath ver 0.5.1. Among the tested antibodies, cGAS, STING, and IFN-α were detected exclusively in the cytoplasm, while γH2AX showed nuclear localization. In contrast, IRF3 exhibited both cytoplasmic and nuclear staining. To quantitatively evaluate IHC staining, the H-score was calculated for each lesion. Each tumor cell detected by pathologists compared with QuPath was assigned one of the following four categories and intensity scores: 0 (no immunostaining), 1+ (weak intensity), 2+ (moderate intensity), and 3+ (strong intensity). The final H-score was the sum of the multiplied value of the intensity score and the percentage of cells showing staining intensity.

In summary, selected regions of interest (ROIs) within the tumor area. The analysis then proceeded with the detection of the cell by choosing a positive cell detection option and setting cell parameters and intensity parameters. The intensity threshold parameters comprised the overall score with three levels of positivity: weak (+1, yellow), moderate (+2, orange), and strong (+3, red). Additionally, an H-score was calculated as part of the final assessment performed by Qu Path analysis, representing the histological score.

### Statistical analysis

Stata v. 18.0 software (Stata Corporation LLC, College Station, USA) was used for all Statistical analyses. The normality of data distribution was assessed using the Shapiro–Wilk test. Since the data were not normally distributed (p < 0.05), comparisons between two groups were performed using the Mann–Whitney U test. The correlation between the expression and the clinicopathological features of CCA patients will be assessed using logistic regression and the chi-squared test was used to compare categorical data. Overall survival analysis will also be performed by determining and comparing median survival times using the Kaplan-Meier analysis and log-rank tests. Cox regression was used for univariate and multivariate analyses. Given the semi-quantitative nature of H-score data and the possibility of tied ranks, Spearman’s rank correlation coefficient was used to evaluate the associations between markers. Statistical significance will be set at p < 0.05 for all data.

## Results

### Demographic data characteristics

This study included 164 patients diagnosed with CCA. The median age was 62, with 41.46% (68 patients) younger than 62 and 58.54% (96 patients) aged 62 or older. The cohort was predominantly male, accounting for 70.73% (116 patients), while 29.27% (48 patients) were female. Tumor size varied among the patients, with 43.34% (76 patients) presenting tumors measuring 5 cm or less, and 53.66% (88 patients) having tumors larger than 5 cm. Based on anatomical location, 23.78% (39 patients) were classified as intrahepatic CCA (iCCA), while 76.22% (125 patients) were extrahepatic CCA (eCCA). Regarding tumor morphology, the most common type was mixed-pattern 39.02% (64 patients), followed by mass-forming 33.53% (55 patients), periductal infiltrating 15.85% (26 patients), and intraductal growing types 11.59% (19 patients). Histologically, 62.20% of the tumors (102 patients) were identified as tubular subtype, while 37.80% (62 patients) were papillary subtype. In terms of tumor differentiation, the majority were well-differentiated 56.70% (93 patients), with 25.00% (41 patients) moderately differentiated, 7.32% (12 patients) poorly differentiated, and 10.98% (18 patients) without available grading data. Lymph node metastasis was observed in 56.71% of the patients (93 patients), while 43.29% (71 patients) showed no lymph node involvement. Lymphovascular invasion was found in 64.63% of patients (106 patients), absent in 24.39% (40 patients), and not reported in 10.98% (18 patients). Perineural invasion was present in 43.90% (72 patients), absent in 8.54% (14 patients), with the remaining 47.56% (78 patients) lacking data on this feature Table 1.

**Table 1:** Demographic data characteristics.

Patient characteristics	n (%)
Age	
< 62	68 (41.46)
> 62	96 (58.54)
Gender	
Male	116 (70.73)
Female	48 (29.27)
Tumor size	
≤ 5 cm	90 (54.88)
> 5 cm	74 (45.12)
Anatomical position	
iCCA	39 (23.78)
eCCA	125 (76.22)
Morphology	
Intraductal growing (ID)	19 (11.59)
Periductal infiltrating (PI)	26 (15.85)
Mass-forming (MF)	55 (33.53)
Mixed	64 (39.02)
Histologic types	
Tubular	102 (62.20)
Papillary	62 (37.80)
Histologic Grade	
Well-differentiated	93 (56.70)
Moderately differentiated	41 (25.00)
Poorly differentiated	12 (7.32)
N/A	18 (10.98)
Lymph node metastasis	
Present	93 (56.71)
Absent	71 (43.29)
Lymphovascular invasion	
Present	106 (64.63)
Absent	40 (24.39)
N/A	18 (10.98)
Perineural invasion	
Present	72 (43.90)
Absent	14 (8.54)
N/A	78 (47.56)

**Abbreviations:** iCCA, intrahepatic cholangiocarcinoma; eCCA, extrahepatic cholangiocarcinoma; N/A, not available.

### Expression Patterns of γH2AX, cGAS, STING, IRF3, and IFN-α in CCA

**Fig 1:**
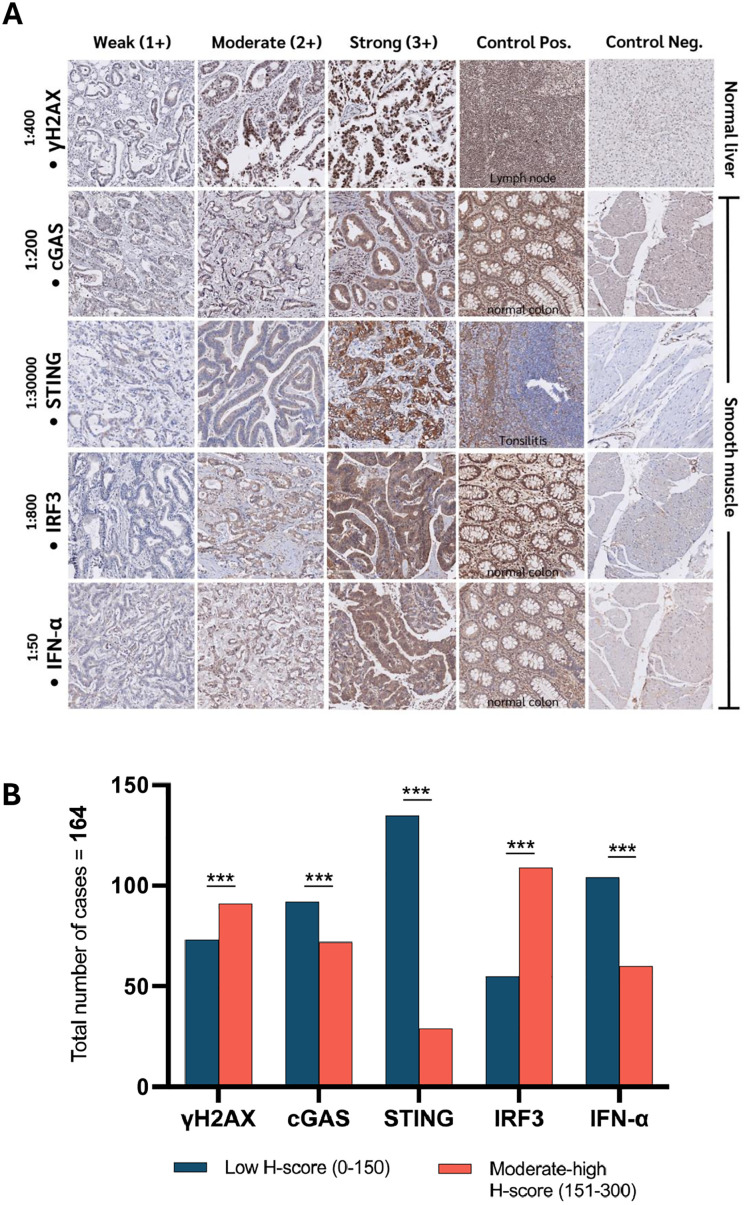
Quantitative immunohistochemical analysis of γH2AX and cGAS–STING pathway markers across the cohort. **(A)** Immunohistochemical staining of γH2AX, cGAS, STING, IRF3, and IFN-α was evaluated across multiple TMA slides and categorized as weak (1+), moderate (2+), or strong (3+). **(B)** Distribution of H-scores for each antibody across 164 cases. H-scores were categorized into two groups: Weak (0–150), Moderate to high (151–300). The Mann–Whitney U test was performed to compare two groups, *p < 0.001. Represents images acquired at 20 × magnification.

The immunohistochemical staining patterns of γH2AX, cGAS, STING, IRF3, and IFN-α in CCA tissues are described. The staining intensity is categorized into three levels: Weak (1+), Moderate (2+), and Strong (3+). Positive and negative controls are also included for validation. γH2AX expression is observed in tumor regions, with lymph node tissue serving as a positive control. cGAS shows varying levels of expression in CCA, with normal colon tissue utilized as a positive control. STING displays expression in some tumor samples, with tonsillitis tissue as a positive control. IRF3 is expressed in CCA, with normal colon tissue serving as a positive control. IFN-α is expressed at different intensities in tumor tissues, with normal colon tissue serving as a positive control. Normal liver and smooth muscle tissues are included as negative controls, confirming specificity. This staining pattern suggests a potential involvement of these markers in tumor biology and immune signaling in CCA, will be shown in Fig 1.

### High cGAS-STING proteins expression associated with clinicopathological features in CCA patients

We investigated correlation between cGAS expression and clinicopathological features in CCA patients. Among various factors, high cGAS expression was significantly associated with smaller tumor size (≤ 5 cm, p = 0.007). Interestingly, the absence of lymph node metastasis tended to exhibit higher levels of cGAS expression (p = 0.030). However, the results showed without of lymphovascular invasion was significantly associated with higher expression of cGAS (p = 0.012). Other variables did not demonstrate statistically significant correlations with cGAS expression (p > 0.05) as shown in a Table 2 Additionally, the correlation between STING expression and clinicopathological features in CCA patients showed that, among the variables, smaller tumor size (≤ 5 cm, p = 0.036) and lymphovascular invasion (p = 0.028) demonstrated a statistically significant association with STING expression. Other clinicopathological variables showed no significant correlation with STING expression (P > 0.05), as shown in Table 3.

**Table 2:** The correlation between cGAS expression and clinicopathological features in CCA patients.

Variables	Category	N = 164	cGAS [n (%)]		
			Low	Moderate-high	*P* value
Age	< 62	68	42 (57.35)	26 (42.65)	0.218
	> 62	96	50 (47.92)	46 (52.08)	
Sex	Male	116	63 (58.33)	53 (41.67)	0.473
	Female	48	29 (49.14)	19 (50.86)	
Tumor size	≤ 5 cm	90	42 (46.67)	48 (53.33)	0.007*
	> 5 cm	74	50 (67.57)	24 (32.43)	
Anatomical positions	iCCA	39	22 (56.82)	17 (43.18)	0.964
	eCCA	125	70 (46.05)	55 (53.95)	
Morphology	ID	19	7 (52.63)	12 (47.37)	0.011*
	PI	26	9 (38.46)	17 (61.54)	
	MF	55	37 (53.70)	18 (46.30)	
	Mixed	64	39 (55.38)	25 (44.62)	
Histology	Papillary	62	37 (68.33)	25 (31.67)	0.471
	Tubular	102	55 (42.31)	47 (57.69)	
Histologic Grade	Well-differentiated	93	50 (51.09)	43 (48.91)	0.246
	Moderately differentiated	41	28 (53.66)	13 (46.34)	
	Poorly differentiated	12	8 (61.54)	4 (38.46)	
Lymph node metastasis	–	71	33 (44.05)	38 (55.95)	0.030*
	+	93	59 (60.00)	34 (40.00)	
Lymphovascular invasion	–	40	16 (32.43)	24 (67.57)	0.012*
	+	106	67 (56.19)	39 (43.81)	
Perineural invasion	–	14	9 (40.00)	5 (60.00)	0.283
	+	72	35 (38.03)	37 (61.97)	

The comparison of categorical variables using the Chi-square test.

*P < 0.05.

**Abbreviations:** cGAS, cyclic GMP-AMP synthase; ID, intraductal growing; MF, mass forming; PI, periductal infiltrating; iCCA, intrahepatic cholangiocarcinoma; eCCA, extrahepatic cholangiocarcinoma.

**Table 3:** The correlation between STING expression and clinicopathological features in CCA patients.

Variables	Category	N = 164	STING [n (%)]		
			Low	Moderate-high	*P* value
Age	< 62	68	59 (55.88)	9 (44.12)	0.209
	> 62	96	76 (54.17)	20 (45.83)	
Sex	Male	116	93 (50.86)	23 (49.14)	0.263
	Female	48	42 (64.58)	6 (35.42)	
Tumor size	≤ 5 cm	90	69 (76.67)	21 (23.33)	0.036*
	> 5 cm	74	66 (89.19)	8 (10.81)	
Anatomical positions	iCCA	39	35 (62.50)	4 (37.50)	0.164
	eCCA	125	100 (46.05)	25 (53.95)	
Morphology	ID	19	14 (63.16)	5 (36.84)	0.236
	PI	26	19 (34.62)	7 (65.38)	
	MF	55	49 (64.81)	6 (35.19)	
	Mixed	64	53 (52.31)	11 (44.62)	
Histology	Papillary	62	52 (68.33)	10 (47.69)	0.684
	Tubular	102	83 (58.33)	19 (47.12)	
Histologic Grade	Well-differentiated	93	76 (52.17)	17 (47.83)	0.980
	Moderately differentiated	41	34 (58.54)	7 (46.34)	
	Poorly differentiated	12	10 (53.85)	62 (41.46)	
Lymph node metastasis	–	71	55 (50.00)	16 (50.00)	0.155
	+	93	80 (60.00)	13 (40.00)	
Lymphovascular invasion	–	40	28 (37.84)	12 (62.16)	0.028*
	+	106	91 (56.19)	15 (43.81)	
Perineural invasion	–	14	13 (60.00)	1 (40.00)	0.228
	+	72	57 (49.30)	15 (50.70)	

The comparison of categorical variables using the Chi-square test.

*P < 0.05; **P < 0.001.

**Abbreviations:** STING, stimulator of interferon genes; ID, intraductal growing; MF, mass forming; PI, periductal infiltrating; iCCA, intrahepatic cholangiocarcinoma; eCCA, extrahepatic cholangiocarcinoma.

### cGAS-STING pathway-related proteins expression level correlated with clinicopathological features in CCA patients

**Fig 2:**
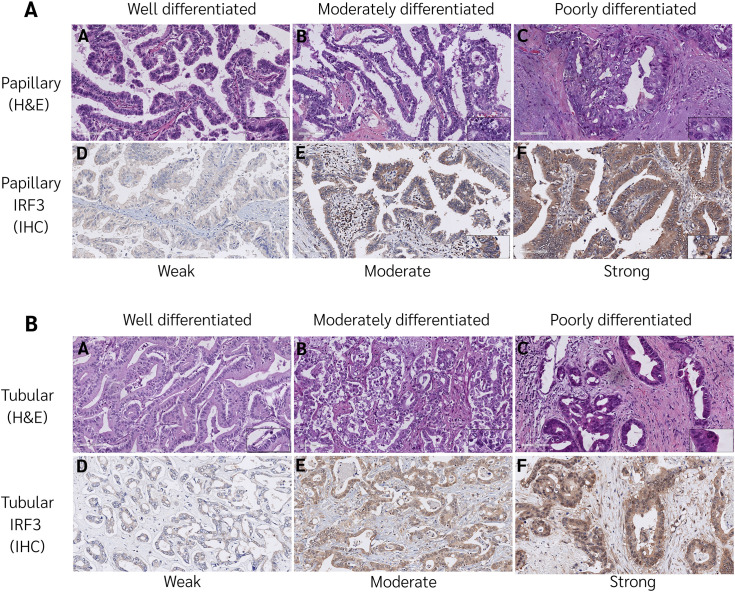
IRF3 expression patterns with histological subtypes of cholangiocarcinoma. **A**. **(A-C)** FFPE from a CCA patients of H&E staining, 20X, scale bar = 100 µm of Well, Moderate and Poorly differentiated papillary subtype. **(D-F)** Weak (1+), Moderate (2+), or Strong (3+) of IRF3 expression patterns. Represents staining, 20X, scale bar = 100 µm. **B**. **(A-C)** FFPE from a CCA patients of H&E staining, 20X, scale bar = 100 µm of Well, Moderate and Poorly differentiated tubular subtype. **(D-F)** Weak (1+), Moderate (2+), or Strong (3+) of IRF3 expression patterns. Represents staining, 20X, scale bar = 100 µm.

To evaluate the association between γH2AX expression and various clinicopathological features in CCA patients, we found that γH2AX expression was significantly associated with lymph node metastasis (p = 0.036) and lymphovascular invasion (p = 0.015). Interestingly, γH2AX expression was significantly higher in patients without perineural invasion (p = 0.014). In contrast, no significant associations were observed between γH2AX expression and other clinicopathological variables (p > 0.05), as shown in Table 4. The correlation between IRF3 expression and clinicopathological features in CCA patients was as shown in Fig 2. IRF3 expression was significantly associated with tumor size exhibited significantly (p = 0.040), anatomical positions (p = 0.048), and histology (p = 0.014) as shown in Table 5. Next, the correlation between IFN-α expression and clinicopathological features in CCA patients was evaluated. None of the clinicopathological variables showed a significant correlation with IFN-α expression (p > 0.05), as shown in Table 6.

**Table 4:** The correlation between γH2AX expression and clinicopathological features in CCA patients.

Variables	Category	N = 164	γH2AX [n (%)]		
			Low	Moderate-high	*P* value
Age	< 62	68	33 (50.00)	35 (50.00)	0.384
	> 62	96	40 (43.75)	56 (56.25)	
Sex	Male	116	51 (44.83)	65 (55.17)	0.827
	Female	48	22 (50.00)	26 (50.00)	
Tumor size	≤ 5 cm	90	38 (42.22)	52 (57.78)	0.515
	> 5 cm	74	35 (47.30)	39 (52.70)	
Anatomical positions	iCCA	39	8 (45.45)	31 (54.55)	0.001*
	eCCA	125	65 (47.37)	60 (52.63)	
Morphology	ID	19	3 (26.32)	16 (73.68)	0.021*
	PI	26	9 (46.15)	17 (53.85)	
	MF	55	27 (53.70)	28 (31.48)	
	Mixed	64	34 (52.31)	30 (47.69)	
Histology	Papillary	62	25(41.67)	37 (58.33)	0.400
	Tubular	102	48 (49.04)	54 (50.96)	
Histologic Grade	Well-differentiated	93	41 (45.65)	52 (54.35)	0.852
	Moderately differentiated	41	20 (48.78)	21 (51.22)	
	Poorly differentiated	12	5 (53.85)	7 (46.15)	
Lymph node metastasis	–	71	25 (38.10)	46 (61.90)	0.036*
	+	93	48 (55.00)	45 (45.00)	
Lymphovascular invasion	–	40	11 (27.03)	29 (72.97)	0.015*
	+	106	53 (51.43)	53 (48.57)	
Perineural invasion	–	14	2 (60.00)	12 (86.67)	0.014*
	+	72	36 (13.33)	36 (49.30)	

The comparison of categorical variables using the Chi-square test.

*P < 0.05.

**Abbreviations:** γH2AX, phosphorylated histone H2AX; ID, intraductal growing; MF, mass forming; PI, periductal infiltrating; iCCA, intrahepatic cholangiocarcinoma; eCCA, extrahepatic cholangiocarcinoma.

**Table 5:** The correlation between IRF3 expression and clinicopathological features in CCA patients.

Variables	Category	N = 164	IRF3 [n (%)]		
			Low	Moderate-high	*P* value
Age	< 62	68	23 (33.82)	45 (66.18)	0.948
	> 62	96	32 (33.33)	64 (66.67)	
Sex	Male	116	40 (34.48)	76 (65.52)	0.690
	Female	48	15 (31.25)	33 (68.75)	
Tumor size	≤ 5 cm	90	24 (26.67)	66 (73.33)	0.040*
	> 5 cm	74	31 (41.89)	43 (58.11)	
Anatomical positions	iCCA	39	8 (20.51)	31 (79.49)	0.048*
	eCCA	125	47 (37.60)	78 (62.40)	
Morphology	ID	19	5 (26.32)	14 (73.68)	0.725
	PI	26	7 (26.72)	19 (73.08)	
	MF	55	20 (36.36)	35 (63.64)	
	Mixed	64	23 (35.94)	41 (64.06)	
Histology	Papillary	62	28 (45.16)	34 (54.84)	0.014*
	Tubular	102	27 (26.47)	75 (73.53)	
Histologic Grade	Well-differentiated	93	32 (34.41)	61 (65.59)	0.459
	Moderately differentiated	41	14 (34.15)	27 (65.85)	
	Poorly differentiated	12	2 (16.67)	10 (83.33)	
Lymph node metastasis	–	71	24 (33.80)	47 (66.20)	0.950
	+	93	31 (33.33)	62 (66.67)	
Lymphovascular invasion	–	40	11 (27.50)	29 (72.50)	0.396
	+	106	37 (34.91)	69 (65.09)	
sss	–	14	7 (50.00)	7 (50.00)	0.195
	+	72	23 (31.94)	49 (68.06)	

The comparison of categorical variables using the Chi-square test

*P < 0.05.

**Abbreviations:** IRF3, interferon regulatory factor 3; ID, intraductal growing; MF, mass forming; PI, periductal infiltrating; iCCA, intrahepatic cholangiocarcinoma; eCCA, extrahepatic cholangiocarcinoma.

**Table 6:** The correlation between IFN-α expression and clinicopathological features in CCA patients.

Variables	Category	N = 164	IFN-α [n (%)]		
			Low	Moderate-high	*P* value
Age	< 62	68	48 (70.59)	20 (29.41)	0.108
	> 62	96	56 (58.33)	40 (41.67)	
Sex	Male	116	75 (64.66)	41 (35.43)	0.608
	Female	48	29 (60.42)	19 (39.58)	
Tumor size	≤ 5 cm	90	54 (60.00)	36 (40.00)	0.317
	> 5 cm	74	50 (67.57)	24 (32.43)	
Anatomical positions	iCCA	39	24 (61.54)	15 (38.46)	0.781
	eCCA	125	80 (64.00)	45 (36.00)	
Morphology	ID	19	13 (68.42)	6 (31.58)	0.857
	PI	26	15 (57.69)	11 (42.31)	
	MF	55	34 (61.82)	21 (38.18)	
	Mixed	64	42 (65.62)	22 (34.38)	
Histology	Papillary	62	41 (66.13)	21 (33.87)	0.574
	Tubular	102	63 (61.76)	39 (38.24)	
Histologic Grade	Well-differentiated	93	56 (60.22)	37 (39.78)	0.601
	Moderately differentiated	41	26 (63.41)	15 (36.59)	
	Poorly differentiated	12	9 (75.00)	3 (25.00)	
Lymph node metastasis	–	71	45 (63.38)	26 (36.62)	0.994
	+	93	59 (63.44)	34 (36.56)	
Lymphovascular invasion	–	40	24 (60.00)	16 (40.00)	0.643
	+	106	68 (64.15)	38 (35.85)	
Perineural invasion	–	14	9 (64.29)	5 (35.71)	0.429
	+	72	38 (52.78)	34 (47.22)	

The comparison of categorical variables using the Chi-square test.

*P < 0.05.

**Abbreviations:** IFN-α, interferon alpha; ID, intraductal growing; MF, mass forming; PI, periductal infiltrating; iCCA, intrahepatic cholangiocarcinoma; eCCA, extrahepatic cholangiocarcinoma.

### Association between cGAS-STING pathway proteins and clinicopathological characteristics in CCA

**Fig 3:**
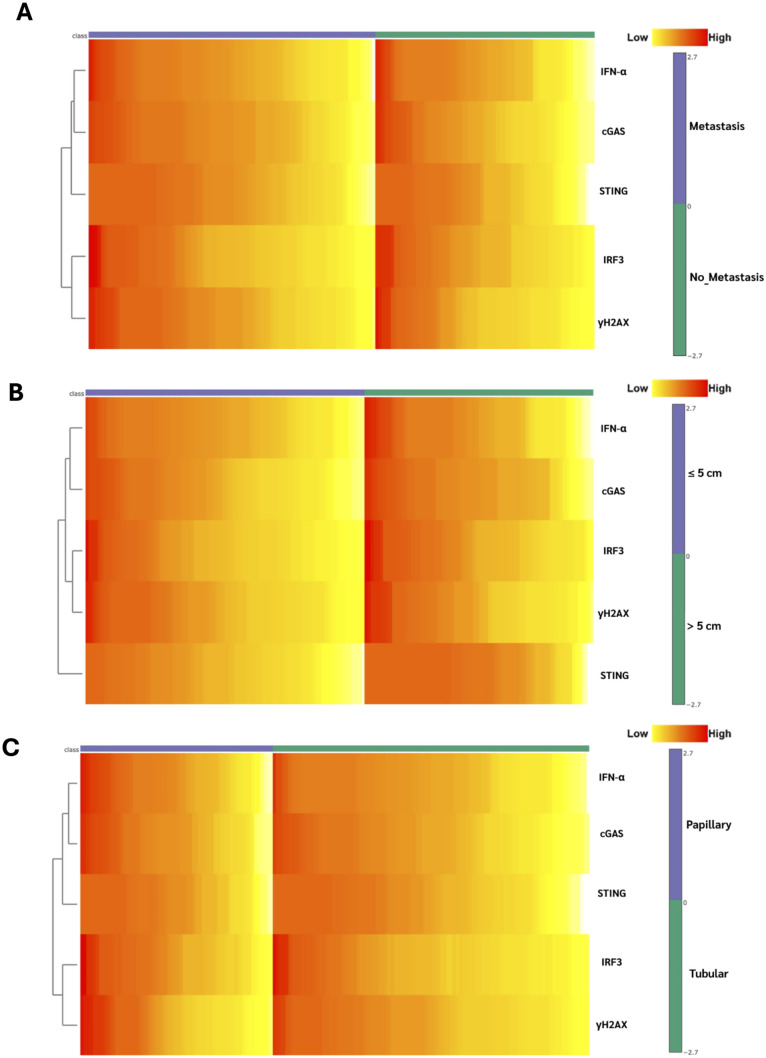
Heatmap visualization of immunohistochemical H-scores (range 0–300) in CCA tissues. (A) lymph node metastasis, (B) tumor size, and (C) histological type.

Association logistic regression revealed significant associations between several cGAS-STING pathway-related proteins and specific clinicopathological features in CCA. Moderate-to-high γH2AX expression was significantly correlated with eCCA exhibiting moderate to high levels of γH2AX have a greater likelihood of improved clinical outcomes (p = 0.001), morphology (p = 0.014). Additionally, γH2AX expression was significantly associated with reduced lymph node metastasis (p = 0.037), lymphovascular invasion (p = 0.016), and perineural invasion (p = 0.025), suggesting a possible protective role against tumor dissemination. Moderate-to-high cGAS expression was significantly correlated with reduced lymph node metastasis (p = 0.031), smaller tumor size (p = 0.008) and lower incidence of lymphovascular invasion (p = 0.016). Similarly, moderate-to-high STING expression was significantly associated with reduced lymphovascular invasion (p = 0.018), and smaller tumor size (p = 0.041). IRF3 expression showed a notable association with smaller tumor size (p = 0.041) and a strong association with the tubular histological subtype of CCA compared to the papillary subtype (p = 0.015), indicating a possible histological subtype preference of IRF3. Other clinicopathological variables showed no significant correlation with IFN-α expression. as shown in Table 7. The heatmap analysis revealed distinct expression patterns of cGAS, STING, IRF3, IFN-α, and γH2AX across clinicopathological subgroups. As shown in Fig 3, In the lymph node metastasis group, cGAS and STING, γH2AX tended to be expressed at higher levels in Fig 3A, compared with the no lymph node metastasis group, whereas STING expression remained relatively comparable between the two. Conversely, IRF3 and IFN-α exhibited relatively higher expression in the no lymph node metastasis group, although the difference from the metastasis group was not pronounced.

**Table 7:** Association analysis of the clinicopathological features and prognostic factors.

Variables	Category	N = 164	Anatomical position (eCCA)		Morphology (Mixed)		Histological type (Tubular)		Histological grade (Poorly)	
			OR (95% CI)	*P*	OR (95% CI)	*P*	OR (95% CI)	*P*	OR (95% CI)	*P*
γH2AX	Low	73	1		1		1	1	1	1
	Moderate-high	91	0.238 (0.101–0.558)	0.001*	0.200 (0.056–0.719)	0.014*	0.760 (0.401–1.440)	0.400	0.883 (0.448–1.737)	0.719
cGAS	Low	92	1		1		1	1	1	1
	Moderate-high	72	1.016 (0.492–2.098)	0.964	0.441 (0.153–1.107)	0.079	1.264 (0.667–2.397)	0.472	0.549 (0.270–1.112)	0.096
STING	Low	135	1		1		1	1	1	1
	Moderate-high	29	2.187 (0.711–6.727)	0.172	0.555 (0.182–1.687)	0.300	1.190 (0.513–2.758)	0.684	0.914 (0.375–2.224)	0.844
IRF3	Low	55	1		1		1	1	1	1
	Moderate-high	109	0.428 (0.181–1.009)	0.053	0.678 (0.231–1.992)	0.480	2.287 (1.175–4.453)	0.015*	1.213 (0.587–2.507)	0.602
IFN-α	Low	104	1		1		1	1	1	1
	Moderate-high	60	0.899 (0.428–1.888)	0.558	1.285 (0.461–3.580	0.631	1.208 (0.624–2.339)	0.574	0.778 (0.384–1.573)	0.485
**Variables**	**Category**	**N = 164**	**Lymph node metastasis**		**Tumor size > 5 cm**		**Lymphovascular invasion**		**Perineural invasion**	
			**OR (95% CI)**	***P***	**OR (95% CI)**	***P***	**OR (95% CI)**	***P***		
γH2AX	Low	73	1		1		1	1	1	1
	Moderate-high	91	0.509 (0.270–0.960)	0.037*	0.814 (0.438–1.512)	0.515	0.379 (0.171–0.837)	0.016*	0.166 (0.034–0.798)	0.025*
cGAS	Low	92	1		1		1	1	1	1
	Moderate-high	72	0.500 (0.266–0.938)	0.031*	0.42 (0.221–0.795)	0.008*	0.388 (0.184–0.817)	0.013*	1.902 (0.580–6.235)	0.288
STING	Low	135	1		1		1	1	1	1
	Moderate-high	29	0.558 (0.248–1.253)	0.158	0.398 (0.164–0.961)	0.041*	0.384 (0.161–0.917)	0.031*	3.421 (0.413–28.273)	0.254
IRF3	Low	55	1		1		1	1	1	1
	Moderate-high	109	1.021 (0.531–1.964)	0.950	0.504 (0.261–0.972)	0.041 *	0.707 (0.317–1.575)	0.397	2.130 (0.668–6.788)	0.201
IFN-α	Low	104	1		1		1	1	1	1
	Moderate-high	60	0.997 (0.525–1.893)	0.994	0.72 (0.378–1.370)	0.317	0.838 (0.397–1.768)	0.643	1.610 (0.491–5.278)	0.431

Association analysis using simple logistic regression.

*P < 0.05; **P < 0.001.

**Abbreviations:** OR Odds ratio;95% CI, 95% confidence interval.

γH2AX, phosphorylated histone H2AX; cGAS, cyclic GMP-AMP synthase; STING, stimulator of interferon genes; IRF3, interferon regulatory factor 3; IFN-α, interferon alpha; ID, intraductal growing; MF, mass forming; PI, periductal infiltrating; iCCA, intrahepatic cholangiocarcinoma; eCCA, extrahepatic cholangiocarcinoma.

Regarding tumor size, the heatmap revealed relatively higher cGAS, STING, and IRF3 expression in tumors > 5 cm, while γH2AX showed no marked changes. IFN-α expression was elevated in both groups, with comparable levels between tumors ≤ 5 cm and > 5 cm in Fig 3B.

Interestingly, IRF3 expression was notably higher in tumors with a tubular histological subtype compared to those with a papillary subtype in Fig 3C, supporting previous statistical findings of a significant association between IRF3 expression and histological type (p = 0.015).

### cGAS-STING pathway-related proteins correlated with clinicopathological parameters and their impact on overall survival in CCA patients

In the univariate analysis, periductal infiltrating (PI) morphology (p = 0.023), poorly differentiated (p = 0.034), lymphovascular invasion (p = 0.036), and moderate–to–high γH2AX expression (p = 0.026) were significantly associated with overall survival (OS) in CCA patients. In the multivariate Cox regression model, moderate–to–high γH2AX expression (p = 0.022), moderate–to–high STING expression (p = 0.038), and a remained independent prognostic factors. Notably, while STING expression was paradoxically associated with poorer survival, despite its previously suggested immune-activating role. These findings underscore the complex prognostic implications of DNA damage and immune signaling markers in CCA shown in Table 8.

**Table 8:** Univariate and multivariate analyses for overall survival time in CCA patients.

Variables	Category	N = 158	Median OS (months)	Univariate analysis		Multivariate analysis	
				HR (95% CI)	*P*	HR (95% CI)	*P*
Age	< 62	65	39.20	1	0.410		
	> 62	93	29.04	1.202 (0.775–1.864)			
Sex	Female	47	25.36	1	0.179	1	0.046
	Male	111	39.20	1.366 (0.866–2.154)		1.718 (1.010–2.922)	
Tumor size	≤ 5	86	30.91	1	0.741		
	> 5	72	29.30	1.075 (0.697–1.660)			
Anatomical positions	iCCA	38	44.42	1	0.218		
	eCCA	120	29.30	1.453 (0.802–2.634)			
Morphology	ID	19	39.20	1		1	
	PI	25	29.04	2.827 (1.157–6.908)	0.023*	3.263 (0.798–13.34)	0.100
	MF	52	23.82	1.959 (0.847–4.529)	0.116	1.472 (0.393–5.509)	0.566
	Mixed	62	30.92	1.844 (0.808–4.204)	0.145	0.997 (0.270–3.680)	0.997
Histology	Tubular	59	39.19	1	0.710		
	Papillary	99	28.71	0.917 (0.584–1.442)			
Histologic Grade	Well-differentiated	92	39.46	1		1	
	Moderately differentiated	39	30.92	1.297 (0.789–2.130)	0.304	1.193 (0.681–2.090)	0.536
	Poorly differentiated	10	21.68	2.186 (1.058–4.517)	0.034*	2.176 (0.983–4.815)	0.055
Lymph node metastasis	–	71	40.18	1	0.142	1	0.333
	+	87	27.40	1.388 (0.896–2.150)		1.317 (0.753–2.302)	
Lymphovascular invasion	–	39	44.91	1	0.036*	1	0.069
	+	101	25.36	1.900 (1.042–3.464)		1.860 (0.952–3.635)	
Perineural invasion	–	14	–	1	0.618		
	+	69	28.71	1.304 (0.459–3.705)			
**Variables**	**Category**	**N = 158**	**Median OS (months)**	**Univariate analysis**		**Multivariate analysis**	
				**HR (95% CI)**	***P***	**HR (95% CI)**	***P***
γH2AX	Low	69	22.14	1	0.026*	1	0.020*
	Moderate-high	89	39.45	0.612 (0.397–9.435)		0.515 (0.293–0.900)	
cGAS	Low	87	26.45	1.102 (0.712–1.706)	0.662	0.865 (0.447–1.672)	0.667
	Moderate-high	71	32.33	1		1	
STING	Low	130	31.50	1		1	0.013*
	Moderate-high	28	29.04	1.300 (0.762–2.219)	0.335	2.308 (1.189–4.480)	
IRF3	Low	52	26.45	1		1	0.346
	Moderate-high	106	31.50	0.868 (0.548–1.375)	0.548	0.749 (0.411–1.365)	
IFN-α	Low	100	39.23	1		1	0.355
	Moderate-high	58	26.45	1.373 (0.880–2.144)	0.162	1.276 (0.761–2.140)	

Univariate and multivariate analysis using Cox’s proportional hazards model.

*P < 0.05.

**Abbreviations:** HR, Hazard ratio;95% CI, 95% confidence interval; OS, Overall survival.

γH2AX, phosphorylated histone H2AX; cGAS, cyclic GMP-AMP synthase; STING, stimulator of interferon genes; IRF3, interferon regulatory factor 3; IFN-α, interferon alpha; ID, intraductal growing; MF, mass forming; PI, periductal infiltrating; iCCA, intrahepatic cholangiocarcinoma; eCCA, extrahepatic cholangiocarcinoma.

### cGAS-STING pathway-related proteins correlated with overall survival in CCA patients

**Fig 4:**
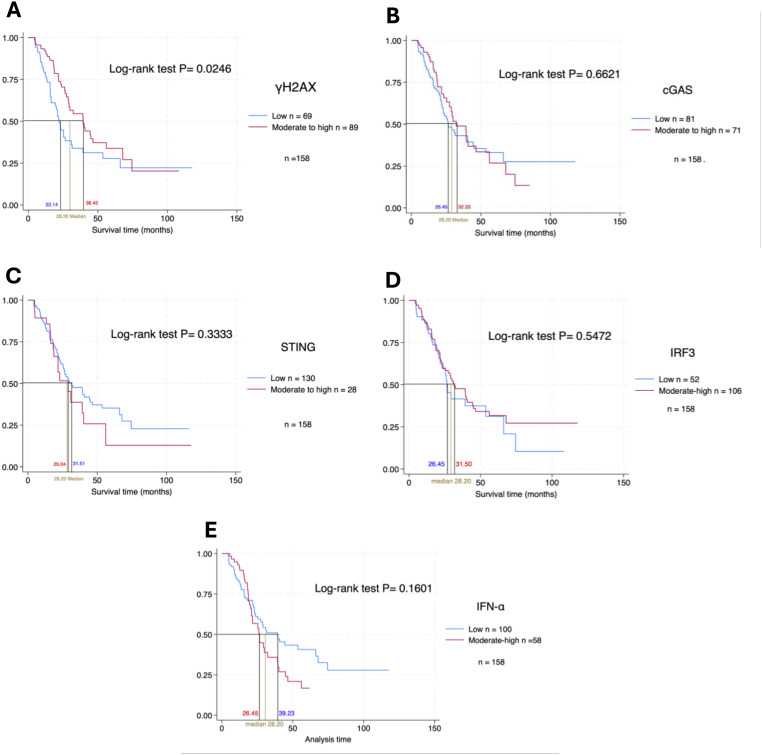
Kaplan–Meier analysis for overall survival in CCA patients stratified by cGAS-STING pathway-related proteins with expression levels. **(A–E)** Kaplan–Meier survival curves comparing patients with low and moderate-to-high expression of γH2AX, cGAS, STING, IRF3, and IFN-α. P-values were calculated using the log-rank test.

Kaplan–Meier survival analysis was performed to assess the prognostic significance of cGAS-STING pathway-related protein expression in CCA patients (Fig 4A–E). Among the proteins evaluated, γH2AX expression showed a statistically significant association with overall survival. Patients with moderate–to–high γH2AX expression had significantly better survival outcomes than those with low expression (p = 0.0246) in Fig 4A. In contrast, no statistically significant differences in survival or other markers in Figs 4B–4E. The median overall survival in the cohort was 28.20 months. STING expression showed 29.04 months in the moderate-high expression group; however, this difference was not statistically significant. Similarly, IRF3 and IFN-α moderate–to–high expression groups showed numerically longer survival, but the results did not reach significance. These findings suggest that γH2AX expression may serve as a potential prognostic marker and may require further investigation with larger sample sizes or alternative analytical approaches.

### Spearman correlation among cGAS-STING pathway-related proteins with expression levels

**Fig 5:**
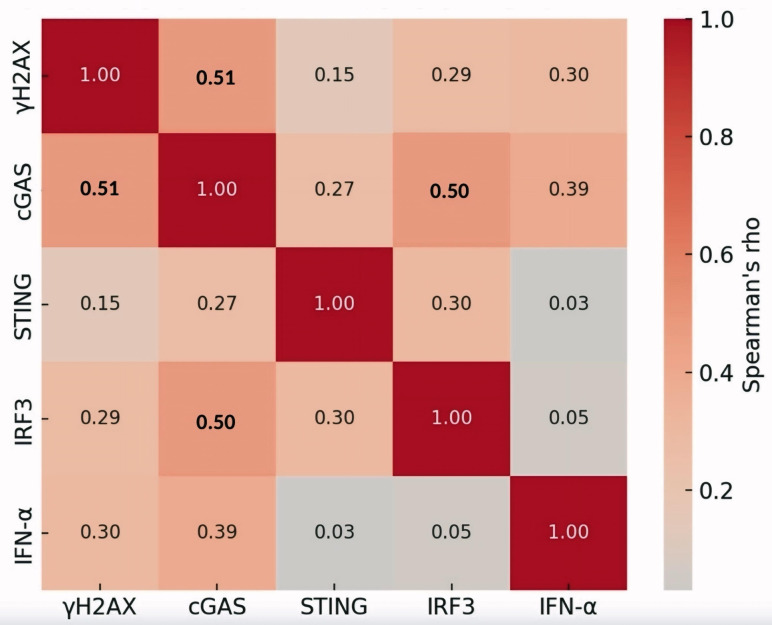
Spearman correlation among cGAS-STING pathway-related proteins with expression levels. The color scale represents the strength and direction of correlation, with numbers in the cells indicating the correlation coefficient (ρ).

The Spearman correlation coefficient was computed to find significant associations between cGAS-STING pathway-related proteins. The median expression level within each subject was used for this calculation. This analysis revealed that the expression of γH2AX and cGAS (ρ = 0.51), cGAS and IRF3 (ρ = 0.50) were moderately associated. Other correlations (ρ < 0.50) were weaker but still showed a moderate or slight relationship, guidance that these proteins may still interact or co-regulate, though the associations are not as strong as shown in Fig 5.

## Discussion

Although the cGAS-STING pathway has been reported to play oncogenic roles in various types of cancer, its expression levels and clinical significance in CCA remain largely unexplored. In this study, we provide evidence that the cGAS-STING pathway is associated with CCA, as the expression of pathway-related proteins—including cGAS, STING, γH2AX, and IRF3—correlates with various clinicopathological features.

CCA is an aggressive malignancy with a poor prognosis. It exhibits marked tumor heterogeneity, meaning that tumors of the same type can vary significantly between patients, and even within the same tumor. This heterogeneity is observed at multiple levels, including genetic, molecular, and cellular, and it profoundly influences tumor behavior, treatment response, and patient outcomes [17].

This study demonstrated a significant downregulation of cGAS expression in CCA patients with lymph node metastasis and vascular invasion, aligning with previous reports that suggest reduced cGAS expression may serve as an immune evasion mechanism of aggressive cancer cells, where alternative DNA detection pathways may promote inflammatory responses and correlate with poor prognosis [18]. The cGAS activates DDR signaling via a STING-TBK1-dependent mechanism, which in turn triggers ATM activation and induces cell cycle arrest, suggesting that cGAS low can increase tumor progression/metastasis [19]. In CCA, the expression of TBK1 was positively correlated with larger tumor diameter, lymph node metastasis, and advanced TNM stage. Functional studies further indicated that TBK1 promotes CCA growth and metastasis both *in vitro* and *in vivo* [7]. With previous research, SRC kinase, often overexpressed in metastatic cancers, phosphorylates and inhibits cGAS, downregulation of which correlates with reduced immune gene expression, and cGAS-STING is associated with higher immune cell infiltration, it may be possible to facilitate tumorigenesis and metastasis by reducing type I IFN production [20–22]. The cGAS-STING pathway, while promoting immune cell recruitment to clear CIN tumors, also plays a role in regulating metastasis [22], suggesting that cGAS downregulation may contribute to enhanced metastasis, this immune checkpoint blockage expression may be an immune escape mechanism [23]. Taken together, they clearly indicate that low cGAS in CCA may contribute to checkpoint evasion, impair DNA repair, and increase tumor aggressiveness.

Interestingly, although moderate-to-high STING expression was associated with a reduced risk of metastasis and invasion in CCA, it was paradoxically correlated with shorter overall survival. This discrepancy may be explained by the time-dependent nature of STING signaling. STING activation may primarily influence early events in tumor progression in Lewis lung carcinoma, brain and colorectal cancer and acute myeloid leukemia [24–27], such as initial immune recognition and suppression of dissemination. However, survival analyses using the log-rank test may fail to capture these early effects, whereas time-sensitive models like the Cox regression with time-by-covariate interaction—particularly those incorporating cubic spline functions—can better account for dynamic hazard risks over time [28]. Another possibility is the presence of confounding clinical or molecular variables in the moderate-to-high STING group. Differences in tumor grade, immune infiltration, or underlying genetic instability could contribute independently to poorer prognosis, regardless of STING activity. While the Cox regression model adjusts for such covariates, the log-rank test does not, which may result in apparent contradictions [29]. In hepatocellular carcinoma, STING expression has been shown to serve as an independent prognostic factor, with its pathway activity influencing tumor behavior and correlating with gene signatures related to the tumor microenvironment [30]. Similarly, our findings in CCA are consistent with studies in melanoma, where STING suppression facilitates immune evasion following DNA damage [31].

Paradoxically, while the statistical analysis demonstrated that moderate-to-high cGAS expression was associated with a reduced risk of lymph node metastasis, the heatmap visualization of H-scores (0–300) revealed an opposite trend: higher cGAS and STING expression tended to appear in the lymph node metastasis group. These contrasting results reflect the multifaceted nature of biomarker analysis. The heatmap may capture global expression tendencies within the study cohort, whereas regression models adjust for multiple covariates and identify independent associations. These contrasting results suggest that although cGAS and STING upregulation may reflect tumor immunogenicity or DNA damage in metastatic cases, their functional consequence could still align with reduced metastatic potential when analyzed in a multivariable context. Therefore, identifying and utilizing alternative protein markers in parallel with STING may offer a more comprehensive understanding of STING activation and its functional relevance [32].

In our study cohort, we observed variable expression levels of cGAS-STING pathway–related proteins. One such protein is γH2AX, which represents one of the earliest cellular responses to DNA double-strand breaks (DSBs) and plays a critical role in maintaining genomic stability [33]. The expression level of γH2AX was significantly higher in cases with lymphovascular invasion and perineural invasion compared to those without invasion. This finding is consistent with a previous study reporting that high γH2AX expression is a poor prognostic factor in oropharyngeal squamous cell carcinoma. Interestingly, γH2AX expression was also significantly elevated in lymph nodes without metastasis. Loss of H2AX promotes EMT characteristics, enhancing cancer cell invasiveness [34]. Conversely, while H2AX is often viewed as a tumor suppressor due to its role in maintaining genomic integrity, its involvement in promoting cancer cell invasiveness through EMT suggests a dual role in tumor biology [35]. Possibly due to cancer treatment often aims to down tumors, which can also reduce the DNA damage linked to cancer. Chemotherapy, such as drugs like poly (ADP-ribose) polymerase inhibitors (PARPis) target DDR pathways, preventing tumor cells from repairing DNA damage effectively [36], reducing tumor burden and the associated DNA damage [37]. Gemcitabine, another chemotherapeutic drug, has demonstrated notable anti-tumor activity. In a pancreatic cancer mouse model, it reduced tumor size by 68% [38]. Moreover, when administered at a low dose alongside radiation therapy, it is associated with a confirmed reduction in tumor size in several cases [39]. Apart from chemotherapy, targeted therapies is another treatment modality that works by blocking specific molecules that drive cancer growth, helping to limit tumor expansion [40]. Expression of IRF3 was significantly high in the tubular histological subtype. IRF3, a key transcription factor in the cGAS–STING pathway, translocates into the nucleus upon activation to induce type I interferons, which are essential for the recruitment of immune cells and the initiation of innate immune responses [41]. However, paradoxically, several studies have reported that inhibition of IRF3 can suppress inflammatory responses and attenuate cell death in non-cancerous tissues. For instance, in tubular epithelial cells, IRF3 has been implicated in the pathogenesis of acute kidney injury, where its inhibition reduces tubulointerstitial inflammation and tissue damage [42]. Similarly, IRF3 was shown to regulate cardiac fibrosis, but not hypertrophy, during angiotensin II-induced hypertension, further highlighting its tissue-specific effects [43]. These findings collectively suggest that while IRF3 generally functions as a pro-inflammatory mediator in immune signaling, its biological role may vary considerably depending on the tissue type, pathological context, and disease state. This tissue-dependent duality of IRF3 activity underscores the complexity of targeting the cGAS–STING–IRF3 axis in therapeutic strategies, particularly in diseases where inflammation and immune responses are finely balanced. In our study cohort, the expression of IFN-α in CCA was not significantly associated with any clinicopathological features. This lack of association may be attributed to several factors, including the distinct biological behavior of CCA and the modulation of immune responses within its tumor microenvironment. Although IFN-α is well recognized for its immunoregulatory and anti-tumor functions, previous studies have reported inconsistent correlations between its expression and clinical outcomes across various cancers. In the context of CCA, the immune landscape appears to be uniquely immunosuppressive and may not mount a robust response to IFN-α signaling. It has been shown that CCA does not significantly enhance the activity of accessory immune cells, suggesting a limited role for IFN-α in modulating immune surveillance and anti-tumor responses in this malignancy [44]. The expression of IFN-α/β receptors in hepatocellular carcinoma does not correlate with improved overall survival or disease-free outcomes, suggesting that receptor expression alone is insufficient to confer therapeutic efficacy [45]. Nevertheless, the mixed tumor profile of CCA, which may exhibit histological and molecular features of both hepatocellular carcinoma and intrahepatic cholangiocarcinoma, complicates the predictive value of IFN-α–based treatments. The therapeutic response to IFN-α may vary considerably depending on the specific characteristics of the tumor, including its cellular origin, differentiation status, and immune microenvironment [46].

This study has certain limitations that should be acknowledged. The sample size, although carefully selected, may not fully capture the heterogeneity of CCA, potentially limiting the generalizability of the findings. The observational design of the study restricts the ability to draw causal conclusions regarding the relationship between cGAS–STING pathway activity and clinical outcomes. While expression levels of pathway-related proteins were correlated with clinicopathological features, the study did not directly assess the functional consequences of such expression changes in cellular or animal models. Moreover, the complexity of the tumor immune microenvironment and intratumoral heterogeneity may influence the observed expression patterns, which could mask underlying biological mechanisms. Additionally, the dynamic and context-dependent roles of molecules like IRF3, γH2AX, and STING were inferred from existing literature, rather than mechanistically validated in the CCA setting.

Although the spearman correlation analysis was based solely on expression protein levels, and the observed associations were relatively weak to moderate, there was still a positive biological trend consistent. This may be attributed to the heterogeneity of clinical samples and the multifactorial regulation of cGAS–STING signaling *in vivo*. Furthermore, this study has certain limitations, including the lack of a healthy control group. This is because obtaining normal bile duct tissues is extremely challenging, as surgical resection is rarely performed in healthy individuals. Nevertheless, the primary aim of this study was to explore the cGAS–STING pathway within the tumor context of cholangiocarcinoma, where its biological significance remains unclear.

## Conclusion

Our findings suggest that STING appears to function as a double-edged sword in CCA, limiting local invasion while paradoxically contributing to poor survival outcomes. IRF3 expression appears linked to histological subtypes, supporting its role in tumor biology. These markers may provide valuable insights into tumor behavior and may guide treatment strategies in CCA patients.

## Abbreviations

CCA: Cholangiocarcinoma
IHC: Immunohistochemistry
CIN: Chromosome instability
dsDNA: double-stranded DNA
DSBs: DNA double-strand breaks
γH2AX: phosphorylated histone H2AX
cGAS: cyclic-GMP-AMP synthase
STING: Stimulator of interferon genes
IRF3: Interferon regulatory factor 3
IFN-α: Interferon alpha
TMA: Tissue microarray
FFPE: Formalin-fixed paraffin-embedded
OS: Overall survival

## References

[1] Sarcognato S, Sacchi D, Fassan M, Fabris L, Cadamuro M, Zanus G, et al. Cholangiocarcinoma. Pathologica. 2021;113(3):158–69. doi: 10.32074/1591-951X-252 34294934 PMC8299326

[2] Yao Y, Dai W. Genomic instability and cancer. J Carcinog Mutagen. 2014;5:1000165. doi: 10.4172/2157-2518.1000165 25541596 PMC4274643

[3] Hanahan D. Hallmarks of cancer: new dimensions. Cancer Discov. 2022;12(1):31–46. doi: 10.1158/2159-8290.CD-21-1059 35022204

[4] Mah L-J, El-Osta A, Karagiannis TC. gammaH2AX: a sensitive molecular marker of DNA damage and repair. Leukemia. 2010;24(4):679–86. doi: 10.1038/leu.2010.6 20130602

[5] Bonner WM, Redon CE, Dickey JS, Nakamura AJ, Sedelnikova OA, Solier S, et al. GammaH2AX and cancer. Nat Rev Cancer. 2008;8(12):957–67. doi: 10.1038/nrc2523 19005492 PMC3094856

[6] Mackenzie KJ, Carroll P, Martin C-A, Murina O, Fluteau A, Simpson DJ, et al. cGAS surveillance of micronuclei links genome instability to innate immunity. Nature. 2017;548(7668):461–5. doi: 10.1038/nature23449 28738408 PMC5870830

[7] Krupina K, Goginashvili A, Cleveland DW. Causes and consequences of micronuclei. Curr Opin Cell Biol. 2021;70:91–9. doi: 10.1016/j.ceb.2021.01.004 33610905 PMC8119331

[8] Chen C, Xu P. Cellular functions of cGAS-STING signaling. Trends Cell Biol. 2023;33(8):630–48. doi: 10.1016/j.tcb.2022.11.001 36437149

[9] Basit A, Cho M-G, Kim E-Y, Kwon D, Kang S-J, Lee J-H. The cGAS/STING/TBK1/IRF3 innate immunity pathway maintains chromosomal stability through regulation of p21 levels. Exp Mol Med. 2020;52(4):643–57. doi: 10.1038/s12276-020-0416-y 32284536 PMC7210884

[10] Sun W, Li Y, Chen L, Chen H, You F, Zhou X, et al. ERIS, an endoplasmic reticulum IFN stimulator, activates innate immune signaling through dimerization. Proc Natl Acad Sci U S A. 2009;106(21):8653–8. doi: 10.1073/pnas.0900850106 19433799 PMC2689030

[11] Kwon J, Bakhoum SF. The cytosolic DNA-sensing cGAS-STING pathway in cancer. Cancer Discov. 2020;10(1):26–39. doi: 10.1158/2159-8290.CD-19-0761 31852718 PMC7151642

[12] Deenonpoe R, Sa-Ngiamwibool P, Watcharadetwittaya S, Thanee M, Intuyod K, Kongpan T, et al. Fluorescence in situ hybridization detection of chromosome 7 and/or 17 polysomy as a prognostic marker for cholangiocarcinoma. Sci Rep. 2022;12(1):8441. doi: 10.1038/s41598-022-11945-8 35589822 PMC9119972

[13] Gan Y, Li X, Han S, Liang Q, Ma X, Rong P, et al. The cGAS/STING pathway: a novel target for cancer therapy. Front Immunol. 2022;12:795401. doi: 10.3389/fimmu.2021.795401 35046953 PMC8761794

[14] Mohseni G, Li J, Ariston Gabriel AN, Du L, Wang Y-S, Wang C. The function of cGAS-STING pathway in treatment of pancreatic cancer. Front Immunol. 2021;12:781032. doi: 10.3389/fimmu.2021.781032 34858438 PMC8630697

[15] Lu C, Guan J, Lu S, Jin Q, Rousseau B, Lu T, et al. DNA sensing in mismatch repair-deficient tumor cells is essential for anti-tumor immunity. Cancer Cell. 2021;39(1):96-108.e6. doi: 10.1016/j.ccell.2020.11.006 33338425 PMC9477183

[16] Abdou P, Wang Z, Chen Q, Chan A, Zhou DR, Gunadhi V, et al. Advances in engineering local drug delivery systems for cancer immunotherapy. Wiley Interdiscip Rev Nanomed Nanobiotechnol. 2020;12(5):e1632. doi: 10.1002/wnan.1632 32255276 PMC7725287

[17] Golino JL, Wang X, Maeng HM, Xie C. Revealing the heterogeneity of the tumor ecosystem of cholangiocarcinoma through single-cell transcriptomics. Cells. 2023;12(6):862. doi: 10.3390/cells12060862 36980203 PMC10047686

[18] Taffoni C, Marines J, Chamma H, Saccas M, Bouzid A, Guha S. The crosstalk between DNA-PK and cGAS drives tumor immunogenicity. bioRxiv. 2022. doi: 2022.06.08.495278

[19] Banerjee D, Langberg K, Abbas S, Odermatt E, Yerramothu P, Volaric M, et al. A non-canonical, interferon-independent signaling activity of cGAMP triggers DNA damage response signaling. Nat Commun. 2021;12(1):6207. doi: 10.1038/s41467-021-26240-9 34707113 PMC8551335

[20] Dunker W, Zaver SA, Pineda JMB, Howard CJ, Bradley RK, Woodward JJ. The proto-oncogene SRC phosphorylates cGAS to inhibit an antitumor immune response. JCI Insight. 2023;8(12):e167270. doi: 10.1172/jci.insight.167270 37166992 PMC10371251

[21] Chen M, Yu S, van der Sluis T, Zwager MC, Schröder CP, van der Vegt B, et al. cGAS-STING pathway expression correlates with genomic instability and immune cell infiltration in breast cancer. NPJ Breast Cancer. 2024;10(1):1. doi: 10.1038/s41523-023-00609-z 38167507 PMC10761738

[22] Dhanisha SS, Guruvayoorappan C. Potential role of cGAS/STING pathway in regulating cancer progression. Crit Rev Oncol Hematol. 2022;178:103780. doi: 10.1016/j.critrevonc.2022.103780 35953012

[23] Sasaki N, Homme M, Kitajima S. Targeting the loss of cGAS/STING signaling in cancer. Cancer Sci. 2023;114(10):3806–15. doi: 10.1111/cas.15913 37475576 PMC10551601

[24] Chen Q, Hong Y, Chen W, Lin F, Zeng J, Huang Y, et al. Prognostic implications of cGAS and STING gene expression in acute myeloid leukemia. Exp Biol Med (Maywood). 2024;249:10108. doi: 10.3389/ebm.2024.10108 38510490 PMC10954193

[25] Chen Q, Boire A, Jin X, Valiente M, Er EE, Lopez-Soto A, et al. Carcinoma-astrocyte gap junctions promote brain metastasis by cGAMP transfer. Nature. 2016;533(7604):493–8. doi: 10.1038/nature18268 27225120 PMC5021195

[26] Lemos H, Mohamed E, Huang L, Ou R, Pacholczyk G, Arbab AS, et al. STING promotes the growth of tumors characterized by low antigenicity via IDO activation. Cancer Res. 2016;76(8):2076–81. doi: 10.1158/0008-5472.CAN-15-1456 26964621 PMC4873329

[27] An X, Zhu Y, Zheng T, Wang G, Zhang M, Li J, et al. An analysis of the expression and association with immune cell infiltration of the cGAS/STING pathway in pan-cancer. Mol Ther Nucleic Acids. 2019;14:80–9. doi: 10.1016/j.omtn.2018.11.003 30583098 PMC6305687

[28] Hess KR. Assessing time-by-covariate interactions in proportional hazards regression models using cubic spline functions. Stat Med. 1994;13(10):1045–62. doi: 10.1002/sim.4780131007 8073200

[29] Bradburn MJ, Clark TG, Love SB, Altman DG. Survival analysis part II: multivariate data analysis--an introduction to concepts and methods. Br J Cancer. 2003;89(3):431–6. doi: 10.1038/sj.bjc.6601119 12888808 PMC2394368

[30] Pu Z, Liu J, Liu Z, Peng F, Zhu Y, Wang X, et al. STING pathway contributes to the prognosis of hepatocellular carcinoma and identification of prognostic gene signatures correlated to tumor microenvironment. Cancer Cell Int. 2022;22(1):314. doi: 10.1186/s12935-022-02734-4 36224658 PMC9554977

[31] Xia T, Konno H, Barber GN. Recurrent loss of STING signaling in melanoma correlates with susceptibility to viral oncolysis. Cancer Res. 2016;76(22):6747–59. doi: 10.1158/0008-5472.CAN-16-1404 27680683

[32] Kim Y, Cho N-Y, Jin L, Jin HY, Kang GH. Prognostic significance of STING expression in solid tumor: a systematic review and meta-analysis. Front Oncol. 2023;13:1244962. doi: 10.3389/fonc.2023.1244962 37711192 PMC10497868

[33] Mah L-J, El-Osta A, Karagiannis TC. gammaH2AX: a sensitive molecular marker of DNA damage and repair. Leukemia. 2010;24(4):679–86. doi: 10.1038/leu.2010.6 20130602

[34] Weyemi U, Redon CE, Sethi TK, Burrell AS, Jailwala P, Kasoji M, et al. Twist1 and Slug mediate H2AX-regulated epithelial-mesenchymal transition in breast cells. Cell Cycle. 2016;15(18):2398–404. doi: 10.1080/15384101.2016.1198864 27315462 PMC5026799

[35] Contreras L, García-Gaipo L, Casar B, Gandarillas A. DNA damage signalling histone H2AX is required for tumour growth. Cell Death Discov. 2024;10(1):99. doi: 10.1038/s41420-024-01869-9 38402225 PMC10894207

[36] Wang M, Chen S, Ao D. Targeting DNA repair pathway in cancer: Mechanisms and clinical application. MedComm (2020). 2021;2(4):654–91. doi: 10.1002/mco2.103 34977872 PMC8706759

[37] Wang Y, Duan M, Peng Z, Fan R, He Y, Zhang H, et al. Advances of DNA damage repair-related drugs and combination with immunotherapy in tumor treatment. Front Immunol. 2022;13:854730. doi: 10.3389/fimmu.2022.854730 35281059 PMC8904426

[38] Bornmann C, Graeser R, Esser N, Ziroli V, Jantscheff P, Keck T, et al. A new liposomal formulation of Gemcitabine is active in an orthotopic mouse model of pancreatic cancer accessible to bioluminescence imaging. Cancer Chemother Pharmacol. 2008;61(3):395–405. doi: 10.1007/s00280-007-0482-z 17554540

[39] Baba H, Suzuki Y, Ema T, Kawakami M, Nakanishi C, Hashimoto T, et al. Gemcitabine concurrent with radiation for locally advanced pancreatic cancer. Gan To Kagaku Ryoho. 2005;32(11):1730–2. 16315923

[40] Joo WD, Visintin I, Mor G. Targeted cancer therapy--are the days of systemic chemotherapy numbered? Maturitas. 2013;76(4):308–14. doi: 10.1016/j.maturitas.2013.09.008 24128673 PMC4610026

[41] Bakhoum SF, Ngo B, Laughney AM, Cavallo J-A, Murphy CJ, Ly P, et al. Chromosomal instability drives metastasis through a cytosolic DNA response. Nature. 2018;553(7689):467–72. doi: 10.1038/nature25432 29342134 PMC5785464

[42] Córdoba-David G, García-Giménez J, Cardoso Castelo-Branco R, Carrasco S, Cannata P, Ortiz A, et al. Crosstalk between TBK1/IKKε and the type I interferon pathway contributes to tubulointerstitial inflammation and kidney tubular injury. Front Pharmacol. 2022;13:987979. doi: 10.3389/fphar.2022.987979 36386242 PMC9647636

[43] Tsushima K, Osawa T, Yanai H, Nakajima A, Takaoka A, Manabe I, et al. IRF3 regulates cardiac fibrosis but not hypertrophy in mice during angiotensin II-induced hypertension. FASEB J. 2011;25(5):1531–43. doi: 10.1096/fj.10-174615 21266535

[44] Ma YT, Zheng L, Zhao CW, Zhang Y, Xu XW, Wang XY, et al. Interferon-α induces differentiation of cancer stem cells and immunosuppression in hepatocellular carcinoma by upregulating CXCL8 secretion. Cytokine. 2024;177:156555. Epub 20240222. doi: 10.1016/j.cyto.2024.156555 38387232

[45] Kondo M, Nagano H, Sakon M, Yamamoto H, Morimoto O, Arai I, et al. Expression of interferon alpha/beta receptor in human hepatocellular carcinoma. Int J Oncol. 2000;17(1):83–8. doi: 10.3892/ijo.17.1.83 10853022

[46] Sucre S, Paredes R, Peters MLB. Immunohistochemical and genetic profiles do not predict behavior of mixed hepatocellular intrahepatic cholangiocarcinoma. American Society of Clinical Oncology. 2023.

